# Quality, chemical composition, and antimicrobial potential of *Citrus* peel essential oils against urinary tract infection pathogens

**DOI:** 10.1038/s41598-026-53016-2

**Published:** 2026-09-14

**Authors:** Farah Aprisza Sheelmarevaa, Pramesthi Reitza Navisya Vasall, Fauzan Setiawan, Nandang Permadi, Jamaludin Al-Anshori, Yaya Rukayadi, Faridah Abas, Euis Julaeha

**Affiliations:** 1 Doctoral Student at Chemistry Study Program, Department of Chemistry, Faculty of Mathematics and Natural Sciences, Universitas Padjadjaran, Jatinangor, 45363 Indonesia; 2 Doctorate Program in Biotechnology, Graduate School, Universitas Padjadjaran, Bandung, 40132 Indonesia; 3 Research Center for Food Crops, Research Organization for Agriculture and Food, National Research and Innovation Agency, Cibinong, 16911 Indonesia; 4 Department of Chemistry, Faculty of Mathematics and Natural Sciences, Universitas Padjadjaran, Jatinangor, 45363 Indonesia; 5 Department of Food Science and Technology, Faculty of Food Science and Technology, Universiti Putra Malaysia, Serdang, 43400 Malaysia

**Keywords:** Antimicrobial, Chemical composition, *Citrus*, Quality, Urinary tract infection, Medical research, Microbiology

## Abstract

Urinary tract infection (UTI) is one of the most common microbial infections worldwide, and the growing resistance to conventional antibiotics highlights the need for alternative therapies. This study evaluated the quality, chemical composition, and antimicrobial activity of *Citrus* peel essential oils (CPEOs) against six UTI-causing pathogens: *Pseudomonas aeruginosa* ATCC 27853, *Klebsiella pneumoniae* ATCC 2537, *Enterococcus faecalis* ATCC 29212, *Staphylococcus aureus* ATCC 6538, *Candida albicans* ATCC 10231, and *Aspergillus niger* ATCC 6275. Essential oils were extracted from the peels of *C. aurantiifolia*, *C. limon*, *C. nobilis*, *C. amblycarpa*, and *C. sinensis*, and their quality parameters were assessed in comparison with ISO standards. The chemical composition determined using gas chromatography–mass spectrometry (GC–MS), revealed limonene, β-pinene, γ-terpinene, and linalool as the predominant constituents, which were further validated by Fourier-transform infrared spectroscopy (FTIR) analysis. Antimicrobial activity was examined using inhibition zone and minimum inhibitory concentration (MIC) assays. The results showed that *C. limon* essential oil (CLEO) exhibited the strongest antimicrobial activity, with inhibition zones reaching up to 81.17 ± 0.827 mm against *K. pneumoniae* and MIC values as low as 2 µg/mL against *C*. *albicans*. In contrast, *C. sinensis* and *C. nobilis* essential oils displayed weaker activity, and no significant inhibition was observed against *P. aeruginosa*. The strong antimicrobial activity of CLEO is likely associated with its higher γ-terpinene content, which is known to disrupt microbial membranes and induce cell death. Overall, these findings highlight CPEOs, particularly CLEO, as promising natural antimicrobial agents for the prevention and management of UTI.

## Introduction

 Urinary tract infection (UTI) is one of the most common infections worldwide and represents a significant cause of morbidity and, in severe cases, mortality. It can affect multiple components of the urinary system, including the urethra, bladder, ureters, and kidneys^1^. Women are particularly susceptible to UTIs due to anatomical and physiological predispositions^2^. Epidemiological data indicate that approximately one-third of women have been diagnosed with a UTI, with 50–70% experiencing at least one episode during their lifetime^3^. UTIs are primarily caused by pathogenic microorganisms such as *Pseudomonas aeruginosa*^4^, *Klebsiella pneumoniae*^5^, and *Enterococcus faecalis*^6^. However, other microorganisms including *Staphylococcus aureus*^7^ and *Candida albicans*^8^ are also implicated, particularly in complicated cases such as catheter-associated, nosocomial, or immunocompromised conditions. In addition, fungal involvement in the urinary tract may occur under specific circumstances, where infections can arise through haematogenous dissemination from systemic aspergillosis or via contiguous spread from adjacent organs^9^. Although *Aspergillus niger* is not a typical uropathogen, its inclusion in this study was intended to provide a broader evaluation of the antifungal potential of the essential oils rather than to represent a primary cause of UTI. Therefore, the microbial panel was designed to include both common uropathogens and selected opportunistic or non-typical organisms to comprehensively assess the antimicrobial spectrum. The exploration of natural antimicrobial agents, particularly from *Citrus* species, offers a promising alternative for the prevention and management of UTI.

The *Citrus* genus, belonging to the Rutaceae family, represents one of the most widely cultivated fruit crops globally due to its high consumption and significant economic value^10^. The growing interest in *Citrus* species is driven by their diverse health-promoting properties, distinctive aroma, and palatable flavor profile^11^. Genus *Citrus* has 16 species, including *C. aurantiifolia*, *C. amblycarpa*, *C. limon*,* C. nobilis*, and *C. sinensis*^12,13^. In addition to their nutritional value, *Citrus* species are notable for producing essential oils composed of complex mixtures of chemical constituents, primarily secondary metabolites such as terpenes and their derivatives, which contribute to their characteristic aroma and diverse biological activities^14,15^. *Citrus* essential oils (CEOs) can be found in various parts of the fruit, particularly concentrated in the peel, which serves as a major reservoir of volatile bioactive compounds^16,17^.

Essential oils (EOs) are aromatic lipophilic liquids synthesized through a series of complex metabolic pathways in plants. EOs in plants serve as a defense mechanism against a wide range of pathogenic microorganisms, including bacteria, fungi, and viruses^18–20^. *Citrus* peel essential oils (CPEOs) have been widely reported to have various activities, including anti-inflammatory^21^, anti-aging^22^, antitumor, anticancer^23^, antibacterial^24^, antifungal^25^, anxiety reducer, and pain reducer^26^. These bioactivities are largely influenced by their chemical composition. CPEOs typically consist of complex mixtures of volatile compounds, predominantly terpenes and their derivatives, such as limonene, β-pinene, sabinene, linalool, α-pinene, myrcene, geraniol, geranial, geranyl acetate, β-caryophyllene, α-terpineol, citronellal, and citronellol^27^. The quality of EOs strongly influences their chemical composition and bioactivity.

Parameters that determine the quality of EOs include refractive index, density, and solubility in alcohol^28^. The refractive index indicates the purity, compound composition, and quality of EOs^29^. Density is the ratio of the density of oil to the density of water. The density value is related to the compound content of the EOs. Solubility in alcohol is closely related to the polarity of the EOs. EOs that contain a higher proportion of polar compounds tend to be more soluble in polar solvents such as ethanol^30^.

This study aimed to evaluate the antimicrobial potential of EOs extracted from the peels of *C. aurantiifolia*,* C. limon*,* C. nobilis*,* C. amblycarpa*, and *C. sinensis* against microorganisms associated with UTIs. Specifically, the study involved screening CPEOs activity against *P. aeruginosa*, *K. pneumoniae*, *E. faecalis*, *S. aureus*, *C. albicans*, and *A. niger*. Prior to antimicrobial testing, the EOs were subjected to quality testing in accordance with ISO standards. Their chemical profiles were then analyzed using gas chromatography–mass spectrometry (GC–MS), and further supported by Fourier-transform infrared spectroscopy (FTIR) analysis to validate the identified functional groups and strengthen the compositional interpretation.

## **Materials and methods**

### Chemical materials

*C. aurantiifolia* peels, *C. amblycarpa* peels, C. *limon* peels, *C. sinensis* peels, and *C. nobilis* peels were obtained from Caringin market, Bandung City, West Java, Indonesia. Plant materials were authenticated by Budi Irawan at Herbarium Jatinangoriense (HJ), Universitas Padjadjaran, Indonesia, under identification letter No. 631/LBM/IT/V/2026, and deposited under the collection reference numbers 768 (*C. limon*), 769 (*C. aurantiifolia*), 770 (*C. amblycarpa*), 771 (*C. sinensis*), and 772 (*C. nobilis*). The chemicals used in this study included distilled water, Na_2_SO_4_, and n-hexane (EMSURE brand).

### CPEO extraction

In this study, CPEOs were obtained using the method described by Julaeha et al. (2023)^31^, with minor modifications. *C. aurantiifolia* peels, *C. amblycarpa* peels, *C. limon* peels, *C. sinensis* peels, and *C. nobilis* peels were distilled using Stahl distillation. The distillation was carried out for 3 h at 150 °C. The resulting EOs were collected then dried over anhydrous Na_2_SO_4_ and finally was stored in a sealed vials in a refrigerator to preserve their quality.

### Quality of CPEOs

Quality evaluation of the distilled CPEOs was conducted by measuring density, refractive index, solubility in alcohol, appearance, color, and aroma. Density of CPEOs were determined using a pycnometer, following the ISO 279:1988 standard, based on Eq. (1):

1 $$\:Density=\:\frac{{m}_{2}-\:{m}_{0}}{{m}_{1}-\:{m}_{0}}$$

where m_0_ is the mass of the empty pycnometer, m_1_ is the mass of the pycnometer filled with distilled water, and m_2_ is the mass of the pycnometer filled with CPEOs.

Alcohol solubility was evaluated based on the procedure described in ISO 875:1999, by gradually adding ethanol of appropriate concentrations into the EOs samples. For appearance and color assessment, the sample vials were placed on a white paper background (beneath and behind the vial) and observed directly. Aroma evaluation was performed by directly smelling the CPEOs.

### Chemical profiling

Chemical profiling of CPEOs was performed using GC–MS. The analysis utilized a DB-35 capillary column as the stationary phase and helium gas as the carrier (mobile) phase. The GC-MS was operated in electron impact (EI) ionization mode at 70 eV, with a mass scan range of *m/z* 50 to 500. The injector and ion source temperatures were set at 250 °C and 280 °C, respectively. The oven temperature program was as follows: initial temperature set at 50 °C for 1 min, increased to 130 °C at a rate of 5 °C/min and held for 0.5 min, then further increased to 250 °C at a rate of 15 °C/min and maintained for 10 min. Helium gas was delivered at a constant flow rate of 1 mL/min.

### FTIR analysis of CPEOs

Functional group of CPEOs was characterized using FTIR connected with a universal ATR (Attenuated Total Retlectance). The spectra were recorded in the range of 4000–400 cm⁻¹ with a resolution of 4 cm⁻¹ and 32 scans per sample. CPEOs were analyzed directly using an ATR. The obtained spectra were analyzed to identify the presence of functional groups based on their characteristic absorption bands.

### Determination of antimicrobial activity of CPEOs

The antimicrobial activity was evaluated against Gram-positive bacteria, namely *S. aureus* (ATCC 6538) and *E. faecalis* (ATCC 29212); Gram-negative bacteria, including *K. pneumoniae* (ATCC 2537) and *P. aeruginosa* (ATCC 27853); and fungal strains, specifically *A. niger* (ATCC 6275) and *C. albicans* (ATCC 10231). All microorganisms were obtained from the Central Laboratory, Universitas Padjadjaran. Antimicrobial activity was assessed using two methods: the inhibition zone assay and the determination of minimum inhibitory concentration (MIC).

The inhibition zone assay was conducted by preparing microbial cultures of each strain and adjusting their concentration to approximately 10⁸ CFU/mL using sterile saline solution. A volume of 500 µL of each microbial suspension was spread onto Mueller-Hinton Agar (MHA) plates using sterile cotton swabs to ensure uniform microbial growth on both control and test plates. Under aseptic conditions, CPEOs were applied onto the agar surface in their undiluted form (100% v/v) to evaluate their direct antimicrobial activity and maximum inhibitory potential. Amoxicillin (0.1%) served as the positive control for *S. aureus*, *K. pneumoniae*, and *P. aeruginosa*. Chlorhexidine (0.2%) was used as the positive control for *E. faecalis*. Ketoconazole (0.5%) and nystatin (0.05%) were used as positive controls for *A. niger* and *C. albicans*, respectively. Distilled water was used as the negative control for the microbial. The petri dishes were incubated at 37 °C for 48 h. Antimicrobial activity was evaluated by measuring the diameter of the inhibition zones (mm).

MIC was determined using the microdilution method. The test substances were first dissolved in 2% dimethyl sulfoxide (DMSO) and then serially two-fold diluted in Mueller Hinton Broth (MHB) using 96-well microplates. Prior to the assay, 2% DMSO was evaluated for antibacterial and antifungal activity and showed no inhibitory effect on the tested microorganisms. Each well was filled with the test substance at varying concentrations, followed by the addition of microbial suspensions. The microplates were then incubated at 37 °C for 48 h. After incubation, microbial growth was assessed based on optical density (OD) measurements. The lowest concentration of the test substance that showed no significant increase in OD, indicating inhibition of microbial growth, was recorded as the MIC.

## Results and discussion

### CPEO extraction

*C. aurantiifolia* essential oil (CAEOs), *C. amblycarpa* essential oil (CCEOs), *C. limon* essential oil (CLEOs), *C. nobilis* essential oil (CNEOs), and *C. sinensis* essential oil (CSEOs) were obtained using the Stahl hydro-distillation method, with yields ranging from 0.14% to 1.08% (Tabel 1). According to Mahato et al. (2019)^32^ and Lin et al. (2019)^33^, essential oil yields generally range between 0.21% and 2.30%, indicating that the yields obtained in this study are within an acceptable and favorable range. As noted by Katekar et al. (2022)^34^ and Ok et al. (2025)^35^, variations in essential oil yield may result from interactions between biological factors (such as plant species, environmental conditions, and harvest time) and technical factors (including extraction methods and processing conditions).

**Table 1:** Yield of CPEOs.

CPEOs	Peel weight (g)	Oil weight (g)	Yield (%)
CAEOs	1860	10.25	0.55 ± 0.056^b^
CCEOs	362	3.89	1.08 ± 0.268^c^
CLEOs	842	4.04	0.48 ± 0.070^ab^
CNEOs	1169.2	1.67	0.14 ± 0.007^a^
CSEOs	742	5.45	0.73 ± 0.014^b^

Note: Values are presented as mean ± SD. Data were analyzed using one-way ANOVA. Different letters within the same column indicate significant differences.

As shown in Tabel 1, CSEOs exhibited the highest yield at 1.08 ± 0.268%, while CNEOs had the lowest yield at 0.14 ± 0.007%. According to Paliwal (2020)^36^, each *Citrus* species possesses distinct physical characteristics of the flavedo, which contribute to the variation in EO yield among different species.

### Quality of CPEOs

Each CPEOs possesses its own unique quality, which can be assessed through parameters such as density, solubility in alcohol, appearance, aroma, and color^37^. To evaluate the conformity of these quality parameters with established standards, the obtained data were compared with ISO specifications, as presented in Table 2.

**Table 2:** Quality of CPEOs.

CPEOs		Density (g/mL)	Solubility in alohol	Refractive index	Aroma	Color
CAEOs	Sample	0.866	1:1	1.475	Lime	Pale Yellow
	ISO 855:2003	0.858–0.866	-	1.474–1.477	Lime	Pale yellow
CCEOs	Sample	0.864	1:1	1.474	Lime	Yellow greenish
	ISO	-	-	-	-	-
CLEOs	Sample	0.853	1:1	1.474	Lemon	Pale yellow
	ISO 855:2003	0.851–0.857	-	1.473–1.475	Lemon	Pale Yellow
CNEOs	Sample	0.847	1:4	1.472	Sweet orange	Pale yellow
	ISO 3528:2012	0.846–0.854	-	1.472–1.475	Sweet orange	Pale yellow
CSEOs	Sample	0.844	1:4	1.472	Sweet orange	Yellow
	ISO 3140:2011	0.842–0.850	-	1.470–1.476	Orange peel	Yellow to greenish

Density is a quantitative characteristic that represents the ratio of the density of the EOs to the density of water at the same temperature and volume. In this study, the measurement was conducted at 20 °C with a volume of 5 mL. The density of extracted CPEOs is range from 0.844 to 0.866 g/mL. As shown in Table 2, density fall within the ISO standard range, indicating that the extracted EOs meet quality standards. According to El-Zaeddi et al. (2017)^38^, variations in density among species are attributed to differences in the chemical composition of each EOs, as each compound possesses its own characteristic density.

Solubility in alcohol is a quantitative characteristic closely related to the polarity of EOs. According to Syahadat el al. (2022)^30^, this parameter is important for assessing the purity of EOs, as oils with high solubility in ethanol are often considered to be of higher quality due to the lower presence of non-volatile impurities or undesirable components. CLEOs exhibited high solubility, with a solubility ratio of 1:1. In contrast, CNEOs and CSEOs showed lower solubility, with 1 mL of EOs dissolving in 4 mL of ethanol.

The refractive index is a quantitative characteristic that reflects the purity, chemical composition, and overall quality of EOs. The refractive indices of CPEOs ranged from 1.472 to 1.475. These values were consistent with the ISO specifications established for each CPEOs. According to Aparco et al. (2023)^29^, EOs with higher refractive index values typically indicate a higher abundance of sesquiterpenes and diterpenes, which are considered desirable components due to their therapeutic properties and overall bioactivity.

**Fig. 1:**
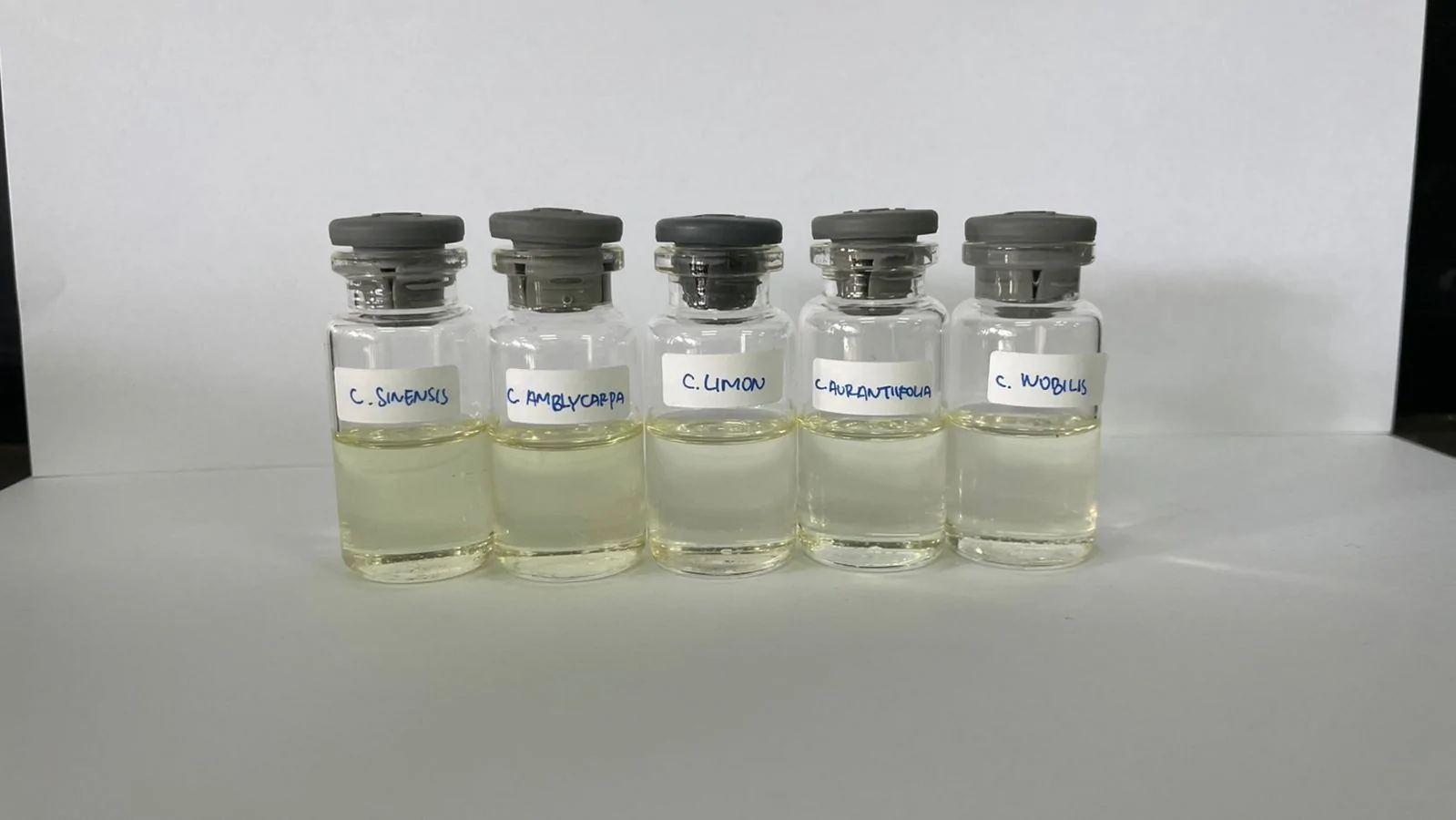
Colors of CPEOs.

Color and aroma are qualitative characteristics of EOs. As shown in Fig. 1, color of the extracted CPEOs conforms to the standards established by ISO. Each EOs exhibited the characteristic *Citrus* aroma, which was also consistent with the ISO specifications for quality and authenticity.

### Chemical profiling

**Fig. 2:**
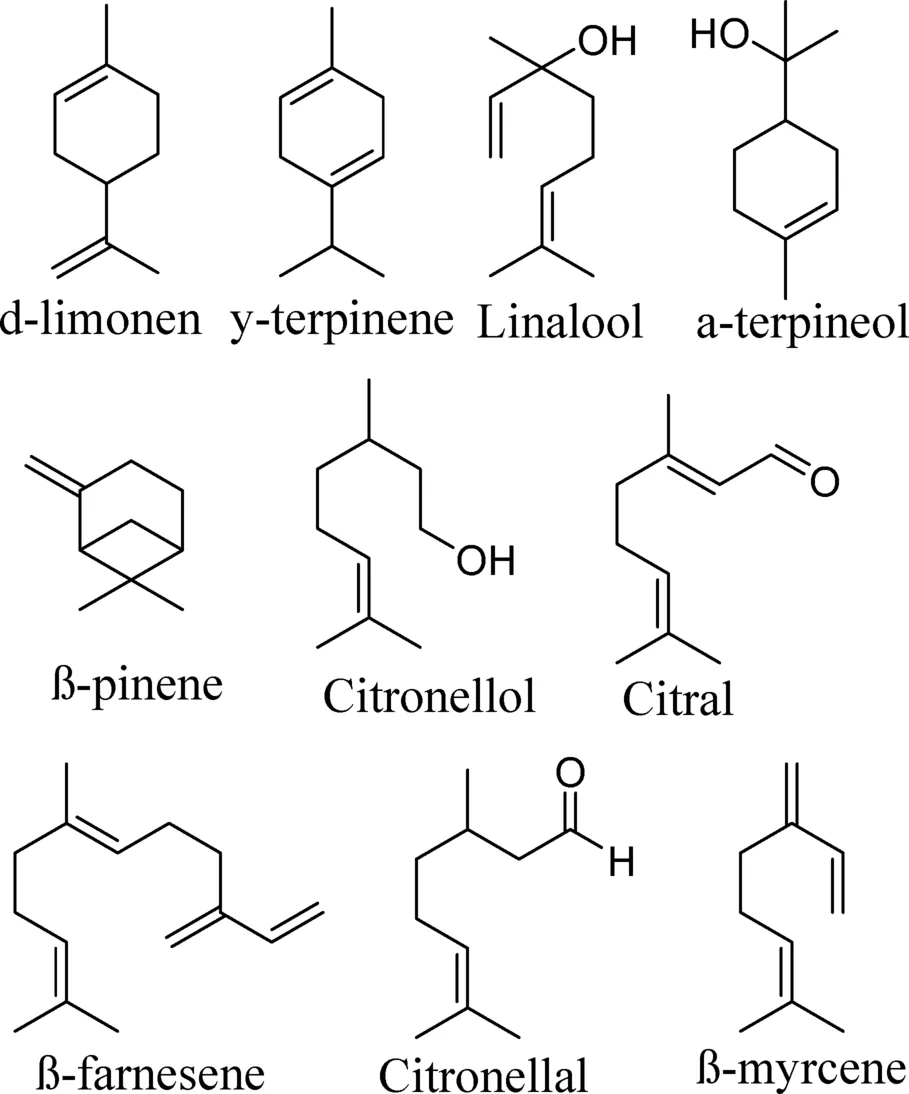
Major components of CPEOs.

The CPEO composition was analyzed using GC–MS. The GC–MS results are summarized in Table 3. The chemical constituents of CPEOs were classified into three groups: monoterpenes, oxygenated monoterpenes, and sesquiterpenes. The major constituents varied among species (Fig. 2). In CAEOs was mainly composed of δ-limonene (28.01%), β-pinene (20.46%), citral (8.99%), and β-farnesene (7.63%). The major component of CCEOs was citronellal (18.29%), β-pinene (17.83%), δ-limonene (11.06%), and citronellol (8.54%). In CLEOs, δ-limonene (54.82%), γ-terpinene (11.34%), β-pinene (3.35%), and α-pinene (2.41%) were the major components. Major components of CNEOs were, δ-limonene (72.92%), β-pinene (13.89%), linalool (4.36%), and β-myrcene (2.20%). Meanwhile, CSEOs were δ-limonene (41.36%), linalool (22.04%), α-terpineol (3.11%), and β-pinene (1.75%). Variations in major constituents may be influenced by several factors, including plant origin, genotype, and environmental conditions such as climate and soil type^27^.

**Table 3:** Chemical composition of CPEOs.

Compound	Formula	Area (%)				
		CAEOs	CCEOs	CLEOs	CNEOs	CSEOs
**Monoterpene**						
α-pinene	C_10_H_16_	0.86	0.66	2.41	1.38	0.37
β-pinene	C_10_H_16_	20.46	17.83	3.35	13.89	1.75
β-myrcene	C_10_H_16_	-	1.32	-	2.2	-
δ-limonene	C_10_H_16_	28.01	11.06	54.82	72.92	41.36
β-ocimene	C_10_H_16_	1.68	-	-	-	0.48
ɣ-terpinene	C_10_H_16_	0.72	1.21	11.34	-	-
o-cymene	C_10_H_14_	-	1.31	1.48	0.47	-
Cosmene	C_10_H_14_	-	-	-	-	0.24
Total		51.73	33.39	73.402	90.86	44.2
**Oxidized Monoterpene**						
Linalool	C_10_H_18_O	1.18	1.17	2.37	4.36	22.04
Nonanal	C_9_H_18_O	-	-	-	0.4	1.01
Oxygenated limonene	C_10_H_16_O	-	-	-	-	0.66
Citronellal	C_10_H_18_O	0.67	18.29	0.74	0.53	1
Terpinen-4-ol	C_10_H_18_O	1.66	2.8	0.5	0.67	0.74
Decanal	C_10_H_20_O	0.59	0.44	-	-	1.05
α-terpineol	C_10_H_18_O	1.36	1.6	0.54	0.79	3.11
Citronellol	C_10_H_20_O	-	8.54	-	-	1.33
Geraniol	C_10_H_18_O	-	0.3	-	-	0.78
Karveol	C_10_H_16_O	-	-	-	-	0.81
Neral	C_10_H_16_O	2.85	-	0.34	-	0.4
δ -karvone	C_10_H_14_O	-	-	-	-	1.51
Citral	C_10_H_16_O	8.99	0.19	0.39	-	0.72
Neril acetate	C_12_H_20_O_2_	0.71	-	-	-	0.28
Geranyl propanoate	C_13_H_22_O_2_	-	-	-	-	0.68
Total		18.01	33.33	4.88	6.75	36.12
**Sesquiterpene**						
β-Farnesene	C_15_H_24_	7.63	-	-	-	-
Caryophyllene	C_15_H_24_	-	0.82	-	-	-
ɣ-elemene	C_15_H_24_	0.8	0.4	-	-	0.98
Humulene	C_15_H_24_	0.64	0.77	-	-	0.53
β-bisabolene	C_15_H_24_	-	-	0.75	-	0.36
α-Farnesene	C_15_H_24_	6.33	1.61	0.45	-	-
Total		15.4	3.6	1.2	0	1.87

CPEOs showed different distributions of chemical classes. The highest monoterpene fraction was observed in CNEOs (90.86%), whereas the lowest was found in CCEOs (33.39%). For oxygenated monoterpenes, CSEOs exhibited the highest proportion (36.12%), while CLEOs showed the lowest (4.88%). The highest sesquiterpene content was detected in CAEOs (15.40%), whereas sesquiterpenes were not detected in CNEOs. These results are consistent with the refractive index data: CAEOs presented the highest refractive index, which aligns with its higher sesquiterpene content.

### FTIR

**Fig. 3:**
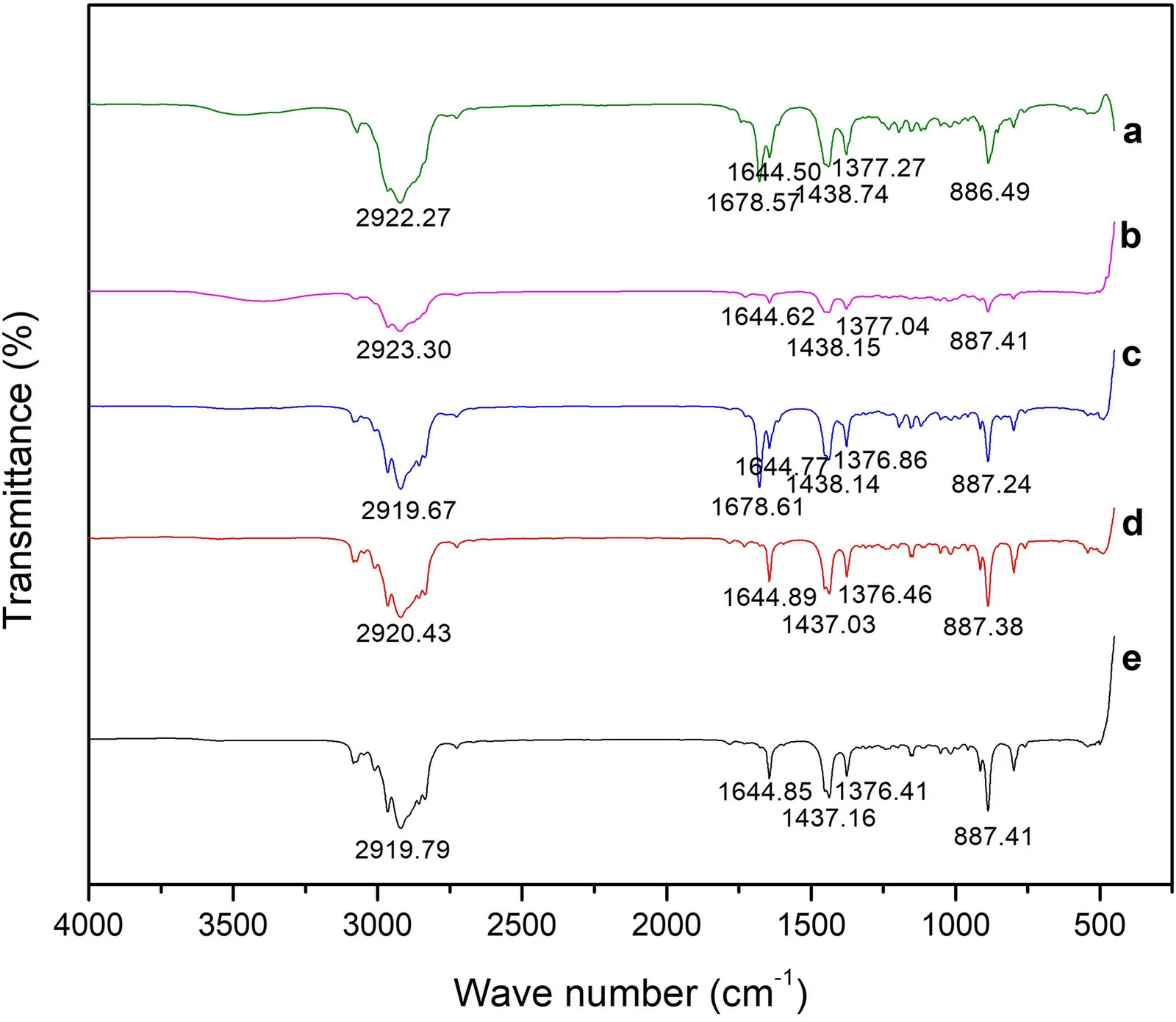
FTIR spectra of (a) CAEOs, (b) CCEOs, (c) CLEOs, (d) CNEOs, (e) CSEOs.

The FTIR spectra of CAEOs, CCEOs, CLEOs, CNEOs, and CSEOs are presented in Fig. 3. The five spectra exhibited similar peak patterns, reflecting the comparable chemical compositions of these EOs. A sharp absorption band at 2919–2920 cm⁻¹ was attributed to C–H stretching vibrations. The band at approximately 1644 cm⁻¹ corresponded to C = C stretching, while the peaks at 1437–1438 cm⁻¹ were assigned to C = CH₂ bending vibrations. In addition, the band at 1376 cm⁻¹ was related to C–H stretching, and the absorption at 889 cm⁻¹ indicated the presence of –CH = CH– bending vibrations. These characteristic bands are commonly associated with terpenoid compounds, which are the major constituents of CEOs.

### Antimicrobial activity

Antimicrobial activity screening was carried out using two approaches: inhibition zone and MIC. This screening aimed to identify the CPEOs with the highest antimicrobial activity against microorganisms associated with UTIs. The assays were performed against six UTI-related microorganisms: *S. aureus*,* E. faecalis*,* K. pneumoniae*,* P. aeruginosa*,* C. albicans*, and *A. niger*. The inhibition zone results are presented in Table 4, and the MIC values are summarized in Table 5.

**Table 4:** Inhibition zone of CPEOs.

Species	CAEOs	CCEOs	CLEOs	CNEOs	CSEOs	Control (+)	Control (-)
**Bacteria (Gram-positive)**							
*Staphylococcus aureus*	24.24 ± 1.393^c^	14.65 ± 0.700^b^	77.26 ± 5.218^e^	8.24 ± 0.282^a^	9.20 ± 0.063^ab^	36.70 ± 0.763^d^	6.00 ± 0^a^
*Enterococcus faecalis*	8.45 ± 0.537^b^	7.24 ± 0.162^a^	9.84 ± 1.004^c^	6.56 ± 0.127^a^	6.56 ± 0.084^a^	29.21 ± 0.197^d^	6.00 ± 0^a^
**Bacteria (Gram-negative)**							
*Klebsiella pneumoniae*	-	56.18 ± 0.664^c^	81.17 ± 0.827^d^	21.45 ± 3.019^b^	13.57 ± 0.042^a^	16.17 ± 0.049^a^	6.00 ± 0^a^
*Pseudomonas aeruginosa*	7.48 ± 0.084^b^	20.62 ± 1.173^d^	6.94 ± 0.120^ab^	6.0 ± 0^a^	6.0 ± 0^a^	18.39 ± 0.183^c^	6.00 ± 0^a^
**Fungi**							
*Candida albicans*	22.96 ± 1.011^c^	28.68 ± 0.254^d^	23.81 ± 0.622^c^	19.07 ± 0.445^b^	10.89 ± 1.301^a^	14.03 ± 0.247^b^	6.00 ± 0^a^
*Aspergillus niger*	38.22 ± 5.402^b^	42.12 ± 7.954^b^	45.97 ± 2.262^b^	35.80 ± 5.366^b^	17.26 ± 0,289^a^	15.86 ± 0.014^a^	6.00 ± 0^a^

Note: Values are presented as mean ± SD. Data were analyzed using one-way ANOVA. Different letters within the same row indicate significant differences.

The qualitative aspects of CPEO composition also influence antimicrobial activity. Ambrosio et al. (2021)^39^ reported that interactions among different compounds can significantly affect overall antimicrobial performance. Although the δ-limonene content in CNEOs (72.92%) is higher than in CLEOs (54.82%), the antimicrobial activity of CLEOs was greater than CNEOs. This may be attributed to the presence of γ-terpinene (11.34%) in CLEOs. Even at relatively low concentrations, γ-terpinene has been reported to exhibit strong and significant antimicrobial activity^40^. The combination of these two compounds may produce synergistic effects that enhance antimicrobial potency^41^. In contrast, although CNEOs contains a higher level of δ-limonene, the presence of linalool may exert antagonistic effects when combined with other constituents^42^. Furthermore, the absence of γ-terpinene in CNEOs may also contribute to its lower antimicrobial activity.

Based on the inhibition zone data obtained from the five *Citrus* species against six microbial strains, CLEOs exhibited the highest activity against four of the tested microorganisms. Accordingly, the subsequent screening step involved determining the MIC values, which are presented in Table 5.

**Table 5:** MIC of CPEOs.

Species	CAEOs	CCEOs	CLEOs	CNEOs	CSEOs
	(µg/mL)				
**Bacteria (Gram-positive)**					
*Staphylococcus aureus*	250.00	250.00	62.00	1000.00	500.00
*Enterococcus faecalis*	20.00	156.00	10.00	5000.00	5000.00
**Bacteria (Gram-negative)**					
*Klebsiella pneumoniae*	62.00	62.00	32.00	1000.00	10000.00
*Pseudomonas aeruginosa*	5000.00	5000.00	5000.00	5000.00	5000.00
**Fungi**					
*Aspergillus niger*	16.00	8.00	16.00	80.00	156.00
*Candida albicans*	4.00	30.00	2.00	30.00	62.00

MIC determination was performed to evaluate the effectiveness of the EOs as antimicrobials against UTI-associated microorganisms. As shown in Table 5, CPEOs exhibited different levels of activity against each microorganism. Overall, MIC data provide a quantitative measure of microbial susceptibility to the EOs, where lower MIC values indicate stronger antimicrobial potency. The MIC values obtained ranged from 2.00 to 5000 µg/mL against the tested Gram-positive bacteria, Gram-negative bacteria, and fungal strains. This indicates a broad-spectrum antimicrobial activity, which is likely influenced by the phytochemical composition of the plant essential oil. In *P. aeruginosa*, the MIC value was notably high, which may be associated with the protective characteristics of the outer membrane in Gram-negative bacteria. This barrier limits the penetration of EOs components, thereby requiring higher concentrations to achieve antimicrobial effects^43^.

The CPEOs showed strong effectiveness against both fungal strains. This may be attributed to the presence of lipophilic bioactive compounds, such as δ-Limonene, which can interact with the fungal cell membrane^44^. GC–MS analysis revealed that CPEOs contain citral, a compound known to inhibit the S-phase of the cell cycle and reduce DNA replication in *C. albicans*. Inhibition of ergosterol synthesis leads to disruption of membrane permeability and integrity. Consequently, cytoplasmic proteins undergo denaturation, and essential cellular processes such as respiration and cell proliferation are impaired. These disruptions prevent the proliferation of *C. albicans* and induce cytomorphometric changes^45^. The combination of these mechanisms contributes to the potent antifungal activity reflected by the low MIC values. Overall, these observations reinforce the potential of CPEOs as natural alternatives for integration into formulations targeting urinary tract infection pathogens.

## Conclusion

This study explored EOs extracted from CAEOs, CCEOs, CLEOs, CNEOs, and CSEOs for their antimicrobial properties. CPEOs were obtained using hydrodistillation, and all five samples produced yields within the acceptable standard range. Furthermore, the CPEOs met the quality requirements of the relevant ISO standards, confirming their suitability as high-quality EOs. The antimicrobial evaluation demonstrated that CPEOs possess notable inhibitory activity against UTI-causing pathogens, with CLEOs showing the strongest effects. The superior activity of CLEOs, as reflected by its large inhibition zones and low MIC values, is likely attributed to its higher γ-terpinene and limonene content, which are known to disrupt microbial cell membranes. In contrast, CSEOs and CNEOs exhibited weaker antimicrobial effects, and none of the samples showed significant activity against *P. aeruginosa*. These findings suggest that CPEOs, particularly CLEOs, have strong potential as natural antimicrobial agents and may serve as complementary alternatives for the prevention and management of UTIs.

## Data Availability

All data generated or analyzed in this study are presented within this article.
